# The distribution and determinants of mammographic density measures in Western Australian aboriginal women

**DOI:** 10.1186/s13058-019-1113-4

**Published:** 2019-02-28

**Authors:** Kirsty McLean, Ellie Darcey, Gemma Cadby, Helen Lund, Leanne Pilkington, Andrew Redfern, Sandra Thompson, Christobel Saunders, Elizabeth Wylie, Jennifer Stone

**Affiliations:** 1 Centre for Genetic Origins of Health and Disease, School of Biomedical Science, Curtin University and The University of Western Australia, Perth, Western Australia Australia; 2 BreastScreen Western Australia, Women and Newborn Health Service, Perth, Western Australia Australia; 3 WA Country Health Service, Government of Western Australia, Perth, Western Australia Australia; 4 School of Medicine, The University of Western Australia, Perth, Western Australia Australia; 5 Fiona Stanley Hospital, Robin Warren Drive, Murdoch, Western Australia Australia; 6 Western Australian Centre for Rural Health, School of Population and Global Health, The University of Western Australia, Geraldton, Western Australia Australia; 7 The Medical Research Foundation, Royal Perth Hospital, Perth, Western Australia Australia; 8 Centre for Genetic Origins of Health and Disease, Curtin University and The University of Western Australia, 35 Stirling Highway M409, Crawley, Western Australia 6009 Australia

**Keywords:** Breast cancer, Risk factor, Mammographic density, Ethnicity, Indigenous women, Mammographic screening

## Abstract

**Background:**

Mammographic density (MD) is an established risk factor for breast cancer. There are significant ethnic differences in MD measures which are consistent with those for corresponding breast cancer risk. This is the first study investigating the distribution and determinants of MD measures within Aboriginal women of Western Australia (WA).

**Methods:**

Epidemiological data and mammographic images were obtained from 628 Aboriginal women and 624 age-, year of screen-, and screening location-matched non-Aboriginal women randomly selected from the BreastScreen Western Australia database. Women were cancer free at the time of their mammogram between 1989 and 2014. MD was measured using the Cumulus software. Kolmogorov-Smirnov tests were used to compare distributions of absolute dense area (DA), precent dense area (PDA), non-dense area (NDA) and total breast area between Aboriginal and non-Aboriginal women. General linear regression was used to estimate the determinants of MD, adjusting for age, NDA, hormone therapy use, family history, measures of socio-economic status and remoteness of residence for Aboriginal and non-Aboriginal women separately.

**Results:**

Aboriginal women were found to have lower DA and PDA and higher NDA than non-Aboriginal women. Age (*p* <  0.001) was negatively associated and several socio-economic indices (p <  0.001) were positively associated with DA and PDA in Aboriginal and non-Aboriginal women. Remoteness of residence was associated with both mammographic measures but for non-Aboriginal women only.

**Conclusions:**

Aboriginal women have, on average, less MD than non-Aboriginal women but the factors associated with MD are similar for both sample populations. Since reduced MD is associated with improved sensitivity of mammography, this study suggests that mammographic screening is a particularly good test for Australian Indigenous women, a population that suffers from high breast cancer mortality.

**Electronic supplementary material:**

The online version of this article (10.1186/s13058-019-1113-4) contains supplementary material, which is available to authorized users.

## Introduction

Mammographic density (MD) is the white radiographic appearance of epithelial and stromal tissue on a mammogram. Increased MD is a strong risk factor for breast cancer [1] and women with dense breasts are more likely to have their cancers missed on mammographic screening due to “masking” and reduced sensitivity [2, 3]. Genetic factors are thought to be a major determinant of MD [4, 5] but it appears that genetic factors primarily determine early life MD and environmental factors regulate later changes [6]. This has important clinical implications for early identification of those at increased risk of breast cancer and/or at increased risk of a cancer going undetected, particularly since MD is modifiable and reducing MD reduces breast cancer risk [7, 8].

There are significant ethnic variations in MD which are consistent with those for breast cancer risk [9] suggesting that there are factors, either genetic, environmental or both, that are common but specific to large populations of women. Several studies have shown that MD measures are lower in South Asians, Japanese, Afro-Caribbean, African Americans, Asian Americans, and higher in Native Hawaiians and New Zealand Maori women compared to European women, consistent with breast cancer incidence rates [9–13]. Little is known about the variation of MD in Aboriginal and Torres Strait Islander women and its association with breast cancer risk factors.

National screening data has shown lower incidence of breast cancer in Aboriginal and Torres Strait Islander women compared to other Australian women [14, 15]. Aboriginal and Torres Strait Islander people make up 2.5% of the Australian population, but their breast cancer mortality rates are two and half times greater than the non-Aboriginal population [16]. Participation in breast cancer screening is significantly lower in Aboriginal and Torres Strait Islander women compared to non-Aboriginal women [17]. Increased information about risk factors like MD and its role in both breast cancer development and detection could help identify and target groups of women that benefit most from mammographic screening, thereby improving early detection and providing better breast cancer outcomes. To this end, the primary aim of this study was to compare the distributions and determinants of MD between Aboriginal and Non-Aboriginal women of Western Australia (WA).

## Methods and materials

### Participants

Within this report, Aboriginal and Torres Strait Islander is used in the national context. Within WA, the term Aboriginal is used in preference to Aboriginal and Torres Strait Islander, in recognition that Aboriginal people are the original inhabitants of WA. No disrespect is intended to our Torres Strait Islander colleagues and community.

Participants were selected from BreastScreen WA, a population based screening program which provides free screening mammograms to all women aged 40 and older and actively targets women aged 50 to 74 years. Seven hundred self-reported Aboriginal women with no personal history of breast cancer and 700 age-, screening year-, and screening location-matched non-Aboriginal women were randomly selected from the BreastScreen WA database via WA’s Department of Health Data Linkage Branch. These women had no history of breast cancer documented by BreastScreen WA and were screened between 1989 and 2014.

### Epidemiological data

Epidemiological data consisted of age, family history of breast cancer (first degree relatives), hormone therapy (HT) (within the 12 months prior to screening), Socio-Economic Indexes for Areas (SEIFA) [18] and Accessibility/Remoteness Index of Australia (ARIA) [19] scores for each participant. There are four SEIFA variables (ecological rather than individual measures) that provide decile rankings of Index of Relative Socio-Economic Disadvantage (henceforth the Disadvantage index), Index of Relative Socio-Economic Advantage and Disadvantage (henceforth the Advantage index), Index of Economic Resources, and Index of Education and Occupation, within a locale. More information regarding these indices can be found in Additional file 1. For the purpose of this study, the decile SEIFA scores were grouped into four categories within each index: 1 (the lowest 10%), 2 (combined deciles of 2, 3 and 4), 3 (combined deciles of 5, 6 and 7) and 4 (combined deciles of 8, 9 and 10). The ARIA score is a tool created by the Australian Bureau of Statistics (ABS) and reflects the degree of remoteness of a particular location by classifying it on a scale of 1–5 (1 - major city, 2 - inner regional, 3 - outer regional, 4 - remote and 5 - very remote).

For the majority of participants, SEIFA and ARIA rankings were estimated using full residential address. If residential information was missing, residential postcode at time of the index mammogram was used to generate SEIFA (*n* = 233) and ARIA (*n* = 212) scores using data from the ABS. If the residential postcode was missing (*n* = 5) the postcode of the fixed screening location was used to generate a SEIFA/ARIA scores. Participants were excluded if we were unable to obtain SEIFA or ARIA scores using these methods (*n* = 26). Sensitivity analyses were performed to compare results both including and excluding women with SEIFA or ARIA information not generated using full residential addresses (See Additional file 1).

### Mammographic data

Film mammograms were digitized using a high powered scanner and copies of full field digital mammograms (FFDM) were extracted from BreastScreen WA’s Picture Archiving and Communication System. All FFDM were produced by the same vendor (Siemens). Of the 1400 women, 86 (6.1%) were excluded due to poor mammographic image quality and 36 (2.1%) were excluded upon discovery of a history of breast cancer via linkage with the WA Cancer Registry. A large portion of subjects (*n* = 260) had had FFDM images.

The Cumulus software [20] was used to estimate absolute dense area (DA), percent dense area (PDA), non-dense area (NDA) and total breast area (TA) from both digitized film and FFDM images. The cranio-caudal view of one (randomly selected) breast side was measured for each women in batches of approximately 100 plus a 10% random repeated sample to check reliability of the observer (JS).

### Statistical methods

STATA IC 14.2 was used for statistical analysis. Correlation coefficients were used to estimate the reliability of the repeated mammographic measurements. Reliability was found to be high (intraclass correlation > 97%) for both DA and PDA. The Kolmogorov-Smirnov test was used to compare breast composition distributions between Aboriginal and non-Aboriginal women. NDA was used as a proxy for body mass index (BMI). Analysis of covariance was used to investigate differences in mean mammographic measures between Aboriginal and non-Aboriginal women adjusting for age and NDA. All stated *P* values were two-sided. Univariate and multivariate regressions were used to investigate the determinants of PDA and DA after square-root transforming each density measure to improve normality of residuals. Age at mammogram and NDA were determined a priori as important determinants of MD and adjusted for in all models. SEIFA and ARIA indices were considered one at a time in all multivariate analyses to avoid collinearity. The final best fitting multivariate regression model was determined using a series of − 2 log likelihood tests and a significance threshold of 0.05.

The final sample included 628 Aboriginal women and 624 Non-Aboriginal women. To avoid systematic differences between MD measures from digitized film and FFDM mammograms, only analyses using the digitized film mammogram data are presented herein (*n* = 992; Aboriginal = 499, Non-Aboriginal = 493) with corresponding estimates from analyses relating to the FFDM image data (*n* = 260; Aboriginal = 129, Non-Aboriginal = 131) provided in Additional file 1.

## Results

Characteristics of study participants are presented in Table 1. The mean age at mammogram was higher for Aboriginal women (53.6 years) than for non-Aboriginal women (52.3 years). The proportion of women with a family history of breast cancer was similar, however HT use was more prevalent within non-Aboriginal women. Aboriginal women were more likely to live in very remote areas and in the lowest 10% for the Advantage index, Disadvantage index, Economic Resources index, and Education and Occupation index than non-Aboriginal women.

**Table 1:** Characteristics of the Aboriginal (*n* = 499) and non-Aboriginal (*n* = 493) women with digitized film images

Characteristics	Aboriginal (*n* = 499)	Non-Aboriginal (*n* = 493)
Mean age at mammogram (SD)	53.6 (8.9)	52.3 (8.5)
HT use in the last 12 months (%)	39 (7.8)	92 (18.7)
Family history of breast cancer (%)	27 (5.4)	40 (8.1)
ARIA^a^ (%)		
Major city	106 (21.2)	129 (26.2)
Inner and outer regional	100 (20.0)	113 (22.9)
Remote	98 (19.6)	111 (22.5)
Very remote	195 (39.1)	140 (28.4)
Advantage and Disadvantage index^b,c^ (%)		
1 (lowest)	215 (43.1)	124 (25.2)
2	175 (35.1)	172 (34.9)
3	82 (16.4)	126 (25.6)
4 (highest)	27 (5.4)	71 (14.4)
Disadvantage index^b,d^ (%)		
1 (lowest)	235 (47.1)	131 (26.6)
2	155 (31.1)	162 (32.9)
3	86 (17.2)	128 (26.0)
4 (highest)	23 (4.6)	72 (14.6)
Economic Resources index^b,e^ (%)		
1 (lowest)	234 (46.9)	132 (26.8)
2	159 (31.9)	165 (33.5)
3	77 (15.4)	131 (26.6)
4 (highest)	29 (5.8)	65 (13.2)
Education and Occupation index^b,f^ (%)		
1 (lowest)	136 (27.3)	64 (13.0)
2	226 (45.3)	181 (36.7)
3	103 (20.6)	156 (31.6)
4 (highest)	34 (6.8)	92 (18.7)
Mean total breast area in cm^2^ (SD)	151.0 (46.5)	123.2 (44.9)
Mean non-dense area in cm^2^ (SD)	138.1 (50.2)	98.4 (49.6)
Mean absolute dense area in cm^2^ (SD)	12.9 (17.2)	24.8 (20.0)
Mean square root absolute dense area (SD)	2.9 (2.1)	4.5 (2.1)
Mean percentage dense area in % (SD)	9.7 (12.5)	22.9 (17.8)
Mean square root percentage dense area (SD)	2.5 (1.9)	4.3 (2.1)

*SD* standard deviation, *HT* hormone therapy

^a^Accessibility/Remoteness Index of Australia (ARIA) scores

^b^SEIFA scores are on a scale from 1 to 4, where 1 indicates the lowest 10% of the population in the state (least advantaged and most disadvantaged, most disadvantaged, least economic resources and least education and occupation opportunities) and 4 indicates the highest 30% of the population in the state (most advantage and least disadvantaged, least disadvantaged, most economic resources and most education/occupation opportunities)

^c^Index of Relative Socio-Economic Advantage and Disadvantage based on Western Australian state rankings

^d^Index of Relative Socio-Economic Disadvantage based on Western Australian state rankings

^e^Index of Economic Resources based on Western Australian state rankings

^f^Index of Education and Occupation based on Western Australian state rankings

Aboriginal women had larger TA (mean = 151.0 cm^2^) and NDA (mean = 138.1 cm^2^) than non-Aboriginal women (TA mean = 123.2 cm^2^; NDA mean = 98.4 cm^2^), however non-Aboriginal women had on average 13.2cm^2^ more DA and 11.9% percentage points higher PDA than Aboriginal women. Analysis of covariance adjusted for age and NDA showed that mean DA and PDA were significantly lower in Aboriginal women (adjusted transformed means = 3.30 and 3.01, respectively) compared to non-Aboriginal women (adjusted transformed means = 4.15 and 3.77, respectively).

**Fig. 1:**
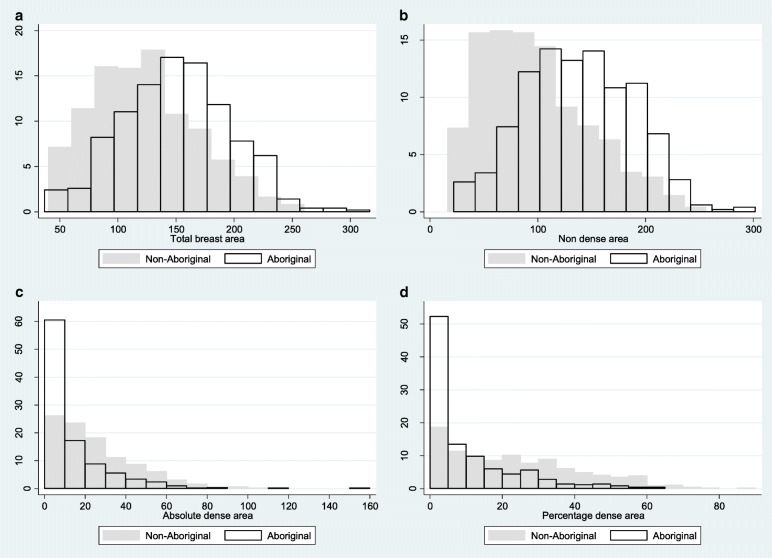
Histograms showing differences in distributions of the total area (**a**), non-dense area (**b**), absolute dense area (**c**) and percentage dense area (**d**) between Aboriginal and non-Aboriginal women for digitized film mammograms across all ages

**Fig. 2:**
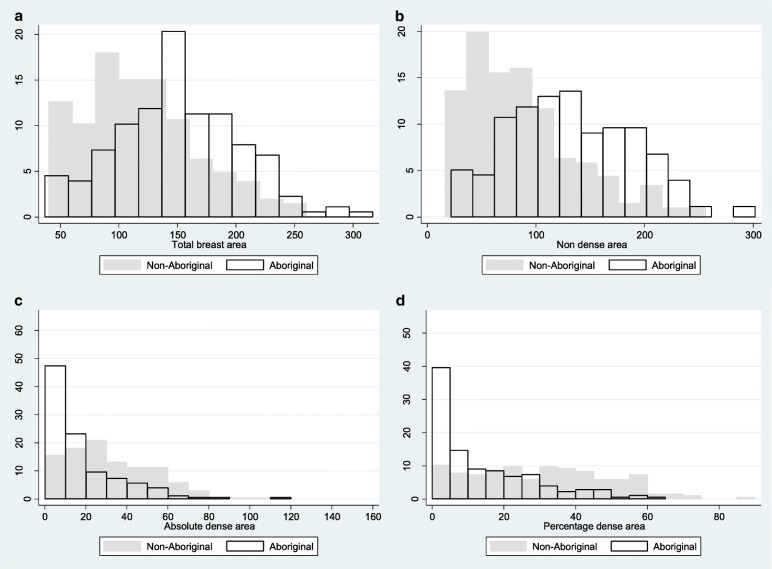
Histograms showing differences in distributions of the total area (**a**), non-dense area (**b**), absolute dense area (**c**) and percentage dense area (**d**) between Aboriginal and non-Aboriginal women aged under 50 for digitized film mammograms

The distributions of the mammographic measures by Aboriginal status are presented in Fig. 1 along with distributions for women younger than 50 years in Fig. 2. The two-sample Kolmogorov-Smirnov test found differences between the distributions of DA and PDA between Aboriginal and non-Aboriginal women (*p* <  0.001).

The regression results for DA are presented in Table 2. Within both Aboriginal and non-Aboriginal women, there was evidence suggesting a negative association between age at mammogram and NDA with DA (both *P* <  0.001). Aboriginal women with a family history of breast cancer had denser breasts than those who had no family history, however there was no evidence of this association in non-Aboriginal women. See Additional file 1: Table S2 for univariate results on FFDM mammograms.

**Table 2:** Univariate and multivariate regression results for absolute dense area among Aboriginal (*n* = 499) and non-Aboriginal (*n* = 493) women with digitized film mammograms

Dense area (cm^2^)^a^	Aboriginal (*n* = 499)				Non-Aboriginal (*n* = 493)			
Characteristic	Univariate		Multivariate		Univariate		Multivariate	
	*β* (SE)	*P* value^b^	*β* (SE)	*P* value^b^	*β* (SE)	*P* value^b^	*β* (SE)	*P* value^b^
Age at mammogram (per year)	− 0.062 (0.010)	< 0.001	*− 0.055 (0.0091)*	*< 0.001*	− 0.078 (0.010)	< 0.001	*− 0.052 (0.0095)*	*< 0.001*
Non-dense area (per cm^2^)	− 0.018 (0.002)	< 0.001	*− 0.017 (0.0016)*	*< 0.001*	− 0.020 (0.0016)	< 0.001	*− 0.018 (0.0016)*	*< 0.001*
HT use in the last 12 months	0.26 (0.35)	0.452			0.062 (0.24)	0.796		
Family history of breast cancer	0.71 (0.41)	0.086	*0.77 (0.36)*	*0.034*	0.048 (0.34)	0.887	*0.11 (0.29)*	*0.693*
ARIA^c^		0.552		0.693		0.014		*0.050*
Major city	Reference		Reference		Reference		*Reference*	
Inner and outer regional	0.17 (0.29)		0.12 (0.25)		− 0.21 (0.26)		*− 0.18 (0.23)*	
Remote	0.22 (0.29)		0.071 (0.26)		0.44 (0.27)		*0.30 (0.23)*	
Very remote	− 0.10 (0.25)		− 0.12 (0.22)		− 0.37 (0.25)		*− 0.29 (0.21)*	
Advantage and Disadvantage index^d,e^		0.147		0.193		0.013		0.176
1 (lowest)	Reference		Reference		Reference		Reference	
2	0.47 (0.21)		0.35 (0.19)		0.37 (0.24)		0.20 (0.21)	
3	0.38 (0.27)		0.25 (0.24)		0.72 (0.26)		0.38 (0.22)	
4 (highest)	0.29 (0.42)		− 0.15 (0.37)		0.83 (0.31)		0.51 (0.26)	
Disadvantage index^d,f^		0.005		0.019		0.075		0.292
1 (lowest)	Reference		Reference		Reference		Reference	
2	0.72 (0.21)		0.57 (0.19)		0.29 (0.24)		0.13 (0.21)	
3	0.56 (0.26)		0.40 (0.23)		0.46 (0.26)		0.25 (0.22)	
4 (highest)	0.61 (0.45)		0.14 (0.40)		0.75 (0.30)		0.47 (0.26)	
Economic Resources index^d,g^		0.016		*0.009*		0.056		0.150
1 (lowest)	Reference		*Reference*		Reference		Reference	
2	0.58 (0.21)		*0.59 (0.19)*		0.50 (0.24)		0.39 (0.20)	
3	0.62 (0.27)		*0.50 (0.24)*		0.64 (0.25)		0.36 (0.22)	
4 (highest)	0.62 (0.41)		*0.18 (0.36)*		0.23 (0.31)		0.033 (0.27)	
Education and Occupation index^d,h^		0.316		0.693		0.001		0.080
1 (lowest)	Reference		Reference		Reference		Reference	
2	0.38 (0.23)		0.23 (0.20)		0.38 (0.30)		0.12 (0.26)	
3	0.41 (0.27)		0.12 (0.24)		0.43 (0.30)		0.24 (0.26)	
4 (highest)	0.41 (0.40)		0.19 (0.35)		1.2 (0.33)		0.64 (0.29)	

SEIFA and ARIA indices were considered one at a time in all multivariate analyses to avoid collinearity. Effect measures in italics were included in the final model. Other effect measures were adjusted for age at mammogram, non-dense area and family history for both Aboriginal and non-Aboriginal women

*SE* standard error, *HT* hormone therapy

^a^Square root transformed

^b^*P* values are based on a − 2 log likelihood test

^c^Accessibility/Remoteness Index of Australia (ARIA) scores

^d^SEIFA scores are on a scale from 1 to 4, where 1 indicates the lowest 10% of the population in the state (least advantaged and most disadvantaged, most disadvantaged, least economic resources and least education and occupation opportunities) and 4 indicates the highest 30% of the population in the state (most advantage and least disadvantaged, least disadvantaged, most economic resources and most education/occupation opportunities)

^e^Index of Relative Socio-Economic Advantage and Disadvantage based on Western Australian state rankings

^f^Index of Relative Socio-Economic Disadvantage based on Western Australian state rankings

^g^Index of Economic Resources based on Western Australian state rankings

^h^Index of Education and Occupation based on Western Australian state rankings

Within Aboriginal women, there was evidence of associations for both the Disadvantage index (*P* = 0.019) and Economic Resources index (*P* = 0.009) with DA. For both indices, DA was higher in the three highest groupings than those in the lowest 10%. Within non-Aboriginal women, there was weak evidence that DA was similarly associated with the Education and Occupation index (*P* = 0.080). In addition, there was marginal evidence of an association between DA and ARIA within non-Aboriginal women (*P* = 0.050), however there was no linear pattern with increasing remoteness. Regression results for PDA, reported in Table 3, are similar to those for DA. See Additional file 1: Table S2 for results for FFDM images which were consistent with those for film images.

**Table 3:** Univariate and multivariate regression results for percentage dense area among Aboriginal (*n* = 499) and non-Aboriginal (*n* = 493) women with digitized film mammograms

Percentage dense area (%)^a^	Aboriginal (*n* = 499)				Non-Aboriginal (*n* = 493)			
Characteristic	Univariate		Multivariate		Univariate		Multivariate	
	*β* (SE)	*P* value^b^	*β* (SE)	*P* value^b^	*β* (SE)	*P* value^b^	*β* (SE)	*P* value^b^
Age at mammogram (per year)	− 0.057 (0.0093)	< 0.001	*− 0.047 (0.0073)*	*< 0.001*	− 0.087 (0.010)	< 0.001	*− 0.043 (0.0070)*	*< 0.001*
Non-dense area (per cm^2^)	− 0.024 (0.0014)	< 0.001	*− 0.023 (0.0013)*	*< 0.001*	− 0.032 (0.0012)	< 0.001	*− 0.029 (0.0012)*	*< 0.001*
HT use in the last 12 months	0.42 (0.32)	0.190			0.089 (0.24)	0.707		
Family history of breast cancer	0.37 (0.38)	0.328			−0.031 (0.34)	0.928		
ARIA^c^		0.458		0.390		0.016		0.052
Major city	Reference		Reference		Reference		Reference	
Inner and outer regional	0.097 (0.27)		0.051 (0.20)		− 0.23 (0.26)		− 0.13 (0.16)	
Remote	0.14 (0.27)		− 0.054 (0.20)		0.42 (0.26)		0.21 (0.17)	
Very remote	− 0.19 (0.23)		− 0.22 (0.18)		− 0.37 (0.25)		− 0.22 (0.16)	
Advantage and Disadvantage index^d,e^		0.074		0.073		0.002		0.075
1 (lowest)	Reference		Reference		Reference		Reference	
2	0.48 (0.20)		0.36 (0.15)		0.53 (0.24)		0.18 (0.15)	
3	0.41 (0.25)		0.29 (0.19)		0.88 (0.26)		0.36 (0.16)	
4 (highest)	0.43 (0.39)		− 0.042 (0.30)		0.91 (0.30)		0.42 (0.19)	
Disadvantage index^d,f^		0.002		0.005		0.021		0.114
1 (lowest)	Reference		Reference		Reference		Reference	
2	0.69 (0.20)		0.52 (0.15)		0.45 (0.24)		0.16 (0.15)	
3	0.52 (0.24)		0.35 (0.18)		0.64 (0.25)		0.30 (0.16)	
4 (highest)	0.82 (0.42)		0.16 (0.32)		0.81 (0.30)		0.41 (0.19)	
Economic Resources index^d,g^		0.020		*0.002*		0.054		0.185
1 (lowest)	Reference		*Reference*		Reference		Reference	
2	0.47 (0.20)		*0.55 (0.15)*		0.49 (0.24)		0.30 (0.15)	
3	0.57 (0.25)		*0.42 (0.19)*		0.67 (0.25)		0.25 (0.16)	
4 (highest)	0.71 (0.38)		*0.21 (0.29)*		0.38 (0.31)		0.088 (0.19)	
Education and Occupation index^d,h^		0.140		0.657		< 0.001		*0.044*
1 (lowest)	Reference		Reference		Reference		*Reference*	
2	0.33 (0.21)		0.19 (0.16)		0.51 (0.29)		*0.092 (0.19)*	
3	0.55 (0.25)		0.18 (0.19)		0.58 (0.30)		*0.24 (0.19)*	
4 (highest)	0.48 (0.37)		0.15 (0.28)		1.4 (0.33)		*0.50 (0.21)*	

SEIFA and ARIA indices were considered one at a time in all multivariate analyses to avoid collinearity. Effect measures in italics were included in the final model. Other effect measures were adjusted for age at mammogram and non-dense area

*SE* standard error, *HT* hormone therapy

^a^Square root transformed

^b^*P* values are based on a − 2 log likelihood test

^c^Accessibility/Remoteness Index of Australia (ARIA) scores

^d^SEIFA scores are on a scale from 1 to 4, where 1 indicates the lowest 10% of the population in the state (least advantaged and most disadvantaged, most disadvantaged, least economic resources and least education and occupation opportunities) and 4 indicates the highest 30% of the population in the state (most advantage and least disadvantaged, least disadvantaged, most economic resources and most education/occupation opportunities)

^e^Index of Relative Socio-Economic Advantage and Disadvantage based on Western Australian state rankings

^f^Index of Relative Socio-Economic Disadvantage based on Western Australian state rankings

^g^Index of Economic Resources based on Western Australian state rankings

^h^Index of Education and Occupation based on Western Australian state rankings

Table 4 shows the percentage of screen-detected and interval-detected cancers by Aboriginal status. The percentage of screen-detected cancers between 2000 and 2016 was slightly higher in non-Aboriginal women compared to Aboriginal women (2.2% vs 2.7%) but the percentage of interval-detected cancers between 2000 and 2014 in non-Aboriginal women was double that of Aboriginal women (0.67% vs 0.32%).

**Table 4:** Screen- vs interval-detected detection percentages by Aboriginal status

	Number of screen-detected breast cancer between 2000 and 2016*	Total number of women screened between 2000 and 2016	Detection percent
Aboriginal women	103	4722	2.18
Non-Aboriginal women	8261	306,595	2.69
	Number of interval-detected breast cancer between 2000 and 2014	Total number of women screened between 2000 and 2014	Detection percent
Aboriginal women	13	4060	0.32
Non-Aboriginal women	1831	275,321	0.67

*Not adjusted for number of screening visits

Characteristics of the participants with FFDM images can be found in Additional file 1: Table S1. Overall, univariate analysis of the FFDM images showed similar patterns to what was found in digitized film results however associations observed were not as robust due to small samples in some categories (Additional file 1: Table S2). Results restricted to women whose SEIFA and ARIA scores were based on full residential address are in Additional file 1: Tables S3 and S4, respectively. Results observed were similar to those found using the whole sample.

## Discussion

We found that Aboriginal women have less MD than non-Aboriginal women but that commonly-reported determinants of MD in European populations are also associated with MD measures in Aboriginal women. We also found that several socio-economic indices are positively associated with MD measures, which is consistent with reports that socio-economic status (SES) is positively associated with breast cancer risk [21–23].

One other study has investigated the distribution of MD in Aboriginal and Torres Strait Islander women [24] and found that they were more likely to have low amounts of MD but did not include a comparable non-Aboriginal sample population and used a visual assessment of parenchymal patterns. Aboriginal women having lower amounts of MD is consistent with lower breast cancer-incidence rates within Aboriginal and Torres Strait Islander women compared to non-Aboriginal women [14, 15]. This provides further evidence of the correlation between population mean MD and breast cancer incidence by ethnicity [9, 25].

The interval-cancer detection rate in Aboriginal women was less than half that of non-Aboriginal women suggesting that Aboriginal women may benefit from improved sensitivity of mammographic screening due to their low breast density. This has significant clinical implications in a population where breast cancer mortality is double that of non-Aboriginal women due to advanced disease [14]. The benefits of increased participation of breast cancer screening within a population that benefits from high mammographic sensitivity but suffers from low survival are potentially great as earlier diagnosis generally equates to less advanced disease and increased survival. Aboriginal women aged 40–49, for whom mammographic screening is free but who are not actively targeted for mammographic screening, may also substantially benefit from mammographic screening due to their lower average density however the absolute risk of Aboriginal women developing breast cancer in this age group is unclear.

Age was a strong determinant of the mammographic measures in both groups of women; consistent with the literature, MD significantly decreases with age [25]. Aboriginal women were found to have larger breasts and more NDA than non-Aboriginal women. Non-dense area is highly correlated with BMI and therefore this result is consistent with higher obesity levels in Aboriginal populations [16]. BreastScreen programs in Australia do not routinely measure height and weight so NDA was used in this study as a proxy for BMI and was strongly negatively associated with both DA and PDA in both groups of women. This is consistent with numerous reports in the literature of strong negative associations of BMI with PDA and, to a lesser extent, DA [26].

Consistent with the literature, HT use was lower in Aboriginal women compared to non-Aboriginal women [27]. HT use is typically positively associated with MD however, the proportion of users was quite small and therefore there was no evidence of association with the MD measures in either group of women. Similarly, family history was marginally higher in non-Aboriginal women compared to Aboriginal women but interestingly was only associated with DA in Aboriginal women despite the small proportion of Aboriginal women with a family history (*n* = 27).

Only a few studies have examined the association of MD with SES or remoteness of living thus far. Aiken and colleagues found that SES was positively associated with PDA but that this association was largely driven by the negative association between SES and BMI [28]. Van der Waal and colleagues also showed that SES was positively associated with volumetric mammographic density but that the potential association between urbanisation and MD was only significant after adjustment for SES [29]. Viel and colleagues used a dichotomous variable of “dependency” as a measure of SES and a dichotomous measure of MD using BIRADS to report that the risk of having dense breasts was lower for women with lower SES status [30]. They also showed marginal evidence of increased density in urban-living women compared to rural-living women. Perry and colleagues also found higher MD levels (BI-RADS) in women living in London relative to those living outside, but did not adjust for reproductive or lifestyle variables [31]. We found that each of the SEIFA measures were associated with both PDA and DA in either Aboriginal or non-Aboriginal women in the univariate analyses. After adjustment for NDA (a proxy for BMI), the evidence of association between most of the SEIFA measures and both measures of MD attenuated in both groups of women. The associations were all positive but not necessarily linear, particularly for Aboriginal women.

There was marginal evidence of an association between remoteness of residence (ARIA) with both mammographic measures but in non-Aboriginal women only (DA: *p* = 0.050; PDA: *p* = 0.052) and the association does not appear to be linear. Due to the matching criteria (age, screening year and screening location), the even distribution of ARIA categories within non-Aboriginal women (Major city 26.2%, Regional 22.9%, Remote 22.5%, Very remote 28.4%) is very different from most other cross-sectional studies of European women which are typically dominated by urban residency. Thus, oversampling women from remote and very remote areas has potentially provided sufficient power to detect the associations between MD and SEIFA/ARIA variables whilst other studies have not.

This study was greatly enhanced by the availability of mammograms and accompanying epidemiological data from a state-wide population-based screening program which allowed for comparisons between SEIFA and ARIA categories. Unfortunately, we did not have access to BMI data. Despite this, previous studies have indicated that NDA is a suitable proxy for BMI [32, 33] and appeared to be an adequate replacement in this study. For example, adjustment for NDA attenuated both the mean differences in the mammographic measures between Aboriginal and non-Aboriginal women and the association between the SEIFA variables and the mammographic measures, indicative of residual confounding due to BMI. A limitation of this study was the high proportion of SEIFA codes generated using residential postcode instead of the optimal complete residential address. It is also known that Aboriginal people experience much higher rates of mobility than non-Aboriginal Australians [34]. However, sensitivity analysis showed similar results using either the appended SEIFA/ARIA data or the SEIFA/ARIA data generated from full residential address only.

Differential effects due to participation bias (in screening and in the study) are unlikely in this study as MD can only be measured from a mammogram and is largely unknown to participants (i.e. unlikely to influence their intention to participate in screening). Finally, the processing applied to FFDM images to improve detection is known to alter the appearance of MD during measurement using the Cumulus software [35]. Despite reduced power to detect associations due to stratification by film/FFDM status, overall interpretation of the results was similar for both datasets; Aboriginal women have, on average, more NDA and lower amounts of MD than other Non-Aboriginal women. The determinants of the mammographic measures were also very similar for the FFDM dataset, providing further validation of the described associations and the robustness of the Cumulus software. However, more versatile software programs that produce validated and comparable mammographic measurements for film/FFDM images would avoid stratification of results.

The present study will serve as a base for future studies and could be used to help inform future mammographic screening practices for Aboriginal women. Culturally-sensitive promotion of the benefits of mammographic screening for Aboriginal women due to lower average density could help improve low participation rates within Aboriginal communities, thereby improving early detection and survival. Recent work has started documenting barriers to mammographic participation and what service providers (and policy makers) need to understand to improve appropriate approaches to breast cancer education and services [36]. BreastScreen WA has several initiatives to improve dissemination of information and participation rates within Aboriginal communities including a Flip Chart as a tool to assist in providing information about breast cancer and screening to Aboriginal women and the Aboriginal Women’s Reference Group which provides BreastScreen direction and support from Aboriginal communities. A concurrent study is investigating the association of MD measures and breast cancer risk in Aboriginal women. Further research is needed to increase understanding as to why Aboriginal women have less MD than other Australian women and how this information can be used to improve breast cancer screening.

## Conclusion

This study found that, in Western Australia, Aboriginal women have, on average, less mammographic density than non-Aboriginal women (and thereby improved sensitivity of mammography) suggesting that mammographic screening is a particularly good test for Australian Indigenous women, a population that suffers from high breast cancer mortality.

## Additional file

Additional file 1 Interpretation of SEIFA variables. **Table S1.** Characteristics of the Aboriginal (*n* = 129) and non-Aboriginal (*n* = 131) women with FFDM mammograms. **Table S2.** Univariate regression results for absolute dense area and percentage dense area among Aboriginal (n = 129) and non-Aboriginal (n = 131) women with FFDM mammograms. **Table S3.** Regression results for absolute dense area among Aboriginal (*n* = 387) and non-Aboriginal (*n* = 430) women with FFDM mammograms where SEIFA and ARIA scores are based on full residential address. **Table S4.** Regression results for percentage dense area among Aboriginal (n = 387) and non-Aboriginal (n = 430) women with FFDM mammograms where SEIFA and ARIA scores are based on full residential address. (DOCX 39 kb)

## Abbreviations

ARIA: Accessibility/Remoteness Index of Australia
BMI: Body mass index
DA: Dense area
FFDM: Full field digital mammogram
HT: Hormone therapy
MD: Mammographic density
NDA: Non-dense area
PDA: Percent dense area
SD: Standard deviation
SE: Standard error
SEIFA: Socio-Economic Indexes for Areas
SES: Socio-economic status
TA: Total area (of the breast)
WA: Western Australia
