# Supplementary material for: The distribution and determinants of mammographic density measures in Western Australian aboriginal women
Source: Breast Cancer Res. 2019 Feb 28;21:33. doi: 10.1186/s13058-019-1113-4 (PMC6393976; doi:10.1186/s13058-019-1113-4)
Supplement: Supplementary file 1 — Interpretation of SEIFA variables. Table S1. Characteristics of the Aboriginal (n = 129) and non-Aboriginal (n = 131) women with FFDM mammograms. Table S2. Univariate regression results for absolute dense area and percentage dense area among Aboriginal (n = 129) and non-Aboriginal (n = 131) women with FFDM mammograms. Table S3. Regression results for absolute dense area among Aboriginal (n = 387) and non-Aboriginal (n = 430) women with FFDM mammograms where SEIFA and ARIA scores are based on full residential address. Table S4. Regression results for percentage dense area among Aboriginal (n = 387) and non-Aboriginal (n = 430) women with FFDM mammograms where SEIFA and ARIA scores are based on full residential address. (DOCX 39 kb) [file 13058_2019_1113_MOESM1_ESM.docx]

**Additional file 1**

**Interpretation of SEIFA variables:** The IRSD (Disadvantage index) summarises a range of information about the economic and social conditions of people and households within an area but includes only measures of relative disadvantage. A **low** score indicates relatively greater disadvantage (e.g. many households with low income, many people with no qualifications, or many people in low skill occupations in general) whilst a **high** score indicates a relative lack of disadvantage (e.g. few households with low incomes, few people with no qualifications, and few people in low skilled occupations).

The IRSAD (Advantage index) summarises information about the economic and social conditions of people and households within an area, including both relative advantage and disadvantage measures (**low** score = low incomes, or many people in unskilled occupations and few households with high incomes, or few people in skilled occupations vs **high** score = in general, opposite).

The IER (Economic Resources index), focuses on the financial aspects of relative socio-economic advantage and disadvantage, by summarising variables related to income and wealth. This index excludes education and occupation variables because they are not direct measures of economic resources (**low** score = low income, or many households paying low rent and few households with high income, or few owned homes vs **high** score = in general, opposite).

The IEO (Education and Occupation index) is designed to reflect the educational and occupational level of communities. The education variables in this index show either the level of qualification achieved or whether further education is being undertaken (**low** score = many people without qualifications, or many people in low skilled occupations or many people unemployed and few people with a high level of qualifications or in highly skilled occupations vs **high** score = in general, opposite).

**Table S1:** Characteristics of the Aboriginal (n=129) and non-Aboriginal (n=131) women with FFDM mammograms.

| Characteristics | Aboriginal (n=129) | Non-Aboriginal (n=131) |
| --- | --- | --- |
| Mean age at mammogram (sd) | 56.1 (8.8) | 56.2 (8.8) |
| HT use in the last 12 months (%) | 4 (3.1) | 10 (7.6) |
| Family history of breast cancer (%) | 9 (7.0) | 20 (15.3) |
| ARIA^a^ (%) |  |  |
| Major city | 51 (39.5) | 57 (43.5) |
| Inner and outer regional | 28 (21.7) | 26 (19.9) |
| Remote | 9 (7.0) | 15 (11.5) |
| Very remote | 41 (31.8) | 33 (25.2) |
| Advantage and disadvantage index^b,c^ (%) |  |  |
| 1 (lowest) | 45 (34.9) | 35 (26.7) |
| 2 | 48 (37.2) | 38 (29.0) |
| 3 | 24 (18.6) | 33 (25.2) |
| 4 (highest) | 12 (9.3) | 25 (19.1) |
| Disadvantage index^b,d^ (%) |  |  |
| 1 (lowest) | 46 (35.7) | 33 (25.2) |
| 2 | 47 (36.4) | 38 (29.0) |
| 3 | 27 (20.9) | 32 (24.4) |
| 4 (highest) | 9 (7.0) | 28 (21.4) |
| Economic resources index^b,e^ (%) |  |  |
| 1 (lowest) | 41 (31.8) | 33 (25.2) |
| 2 | 46 (35.7) | 30 (22.9) |
| 3 | 26 (20.2) | 33 (25.2) |
| 4 (highest) | 16 (12.4) | 35 (26.7) |
| Education and occupation index^b,f^ (%) |  |  |
| 1 (lowest) | 24 (18.6) | 10 (7.6) |
| 2 | 61 (47.3) | 54 (41.2) |
| 3 | 35 (27.1) | 44 (33.6) |
| 4 (highest) | 9 (7.0) | 23 (17.6) |
| Mean total area in cm^2^ (sd) | 150.5 (85.2) | 117.6 (57.9) |
| Mean non-dense area cm^2^ (sd) | 143.9 (81.8) | 109.7 (56.1) |
| Mean percentage dense area in % (sd) | 4.7 (7.1) | 7.5 (9.3) |
| Mean square root percentage dense area (sd) | 1.6 (1.5) | 2.3 (1.6) |
| Mean absolute dense area in cm^2^ (sd) | 6.6 (12.0) | 7.8 (10.2) |
| Mean square root absolute dense area (sd) | 1.9 (1.7) | 2.3 (1.5) |

1. Accessibility/Remoteness Index of Australia (ARIA) scores
2. SEIFA scores are on a scale from 1 to 4, where 1 indicates the lowest 10% of the population in the state (least advantaged and most disadvantaged, most disadvantaged, least economic resources and least education and occupation opportunities) and 4 indicates the highest 30% of the population in the state (most advantage and least disadvantaged, least disadvantaged, most economic resources and most education/occupation opportunities).
3. Index of Relative Socio-Economic Advantage and Disadvantage based on Western Australian state rankings
4. Index of Relative Socio-Economic Disadvantage based on Western Australian state rankings
5. Index of Economic Resources based on Western Australian state rankings
6. Index of Education and Occupation based on Western Australian state rankings

**Abbreviations:** sd, standard deviation; HT, Hormone Therapy; FFDM, Full Field Digital Mammograms

**Table S2:** Univariate regression results for absolute dense area and percentage dense area among Aboriginal (n=129) and non-Aboriginal (n=131) women with FFDM mammograms.

| Characteristic | Absolute Dense Area^a^ | | | | Percentage Dense Area^a^ | | | |
| --- | --- | --- | --- | --- | --- | --- | --- | --- |
|  | Aboriginal (n=129) | | Non-Aboriginal (n=131) | | Aboriginal (n=129) | | Non-Aboriginal (n=131) | |
|  | β (SE) | P-value^b^ | β (SE) | P-value^b^ | β (SE) | P-value^b^ | β (SE) | P-value^b^ |
| Age at mammogram (per year) | -0.078 (0.015) | <0.001 | -0.058 (0.015) | <0.001 | -0.070 (0.014) | <0.001 | -0.060 (0.015) | <0.001 |
| Non-dense area (per cm^2^) | 0.0017 (0.0018) | 0.347 | -0.00051 (0.0024) | 0.835 | -0.0041 (0.0016) | 0.011 | -0.011 (0.0023) | <0.001 |
| HT use in the last 12 months | -0.32 (0.85) | 0.705 | 0.12 (0.51) | 0.817 | -0.18 (0.76) | 0.814 | 0.42 (0.52) | 0.419 |
| Family history of breast cancer | -0.11 (0.58) | 0.850 | -0.13 (0.38) | 0.739 | 0.011 (0.52) | 0.983 | -0.068 (0.38) | 0.859 |
| ARIA^c^ |  | 0.048 |  | 0.402 |  | 0.210 |  | 0.354 |
| Major city | Reference |  | Reference |  | Reference |  | Reference |  |
| Inner and outer regional | 0.72 (0.39) |  | 0.094 (0.37) |  | 0.46 (0.35) |  | -0.35 (0.37) |  |
| Remote | -0.56 (0.59) |  | -0.016 (0.45) |  | -0.41 (0.54) |  | -0.24 (0.46) |  |
| Very remote | -0.32 (0.34) |  | -0.50 (0.34) |  | -0.24 (0.31) |  | -0.60 (0.34) |  |
| Advantage and disadvantage index^d,e^ |  | 0.379 |  | 0.002 |  | 0.414 |  | 0.003 |
| 1 (lowest) | Reference |  | Reference |  | Reference |  | Reference |  |
| 2 | 0.52 (0.35) |  | 0.78 (0.35) |  | 0.45 (0.31) |  | 0.81 (0.35) |  |
| 3 | 0.61 (0.42) |  | 0.71 (0.36) |  | 0.51 (0.38) |  | 0.77 (0.37) |  |
| 4 (highest) | 0.34 (0.54) |  | 1.5 (0.39) |  | 0.21 (0.49) |  | 1.5 (0.39) |  |
| Disadvantage index^d,f^ |  | 0.146 |  | <0.001 |  | 0.175 |  | <0.001 |
| 1 (lowest) | Reference |  | Reference |  | Reference |  | Reference |  |
| 2 | 0.64 (0.34) |  | 0.77 (0.35) |  | 0.57 (0.31) |  | 0.82 (0.35) |  |
| 3 | 0.68 (0.40) |  | 0.70 (0.36) |  | 0.53 (0.36) |  | 0.70 (0.37) |  |
| 4 (highest) | -0.15 (0.61) |  | 1.6 (0.38) |  | -0.13 (0.54) |  | 1.6 (0.38) |  |
| Economic resources index^d,g^ |  | 0.202 |  | 0.010 |  | 0.106 |  | 0.012 |
| 1 (lowest) | Reference |  | Reference |  | Reference |  | Reference |  |
| 2 | 0.13 (0.36) |  | 0.35 (0.38) |  | 0.063 (0.32) |  | 0.43 (0.38) |  |
| 3 | 0.70 (0.42) |  | 0.60 (0.37) |  | 0.78 (0.37) |  | 0.66 (0.38) |  |
| 4 (highest) | 0.76 (0.49) |  | 1.2 (0.36) |  | 0.60 (0.44) |  | 1.2 (0.37) |  |
| Education and occupation index^d,h^ |  | 0.149 |  | 0.625 |  | 0.039 |  | 0.581 |
| 1 (lowest) | Reference |  | Reference |  | Reference |  | Reference |  |
| 2 | 0.80 (0.40) |  | 0.54 (0.53) |  | 0.89 (0.35) |  | 0.53 (0.54) |  |
| 3 | 0.89 (0.44) |  | 0.61 (0.54) |  | 0.93 (0.39) |  | 0.62 (0.55) |  |
| 4 (highest) | 1.01 (0.65) |  | 0.75 (0.59) |  | 1.26 (0.57) |  | 0.81 (0.60) |  |

^a^Square root transformed; ^b^p values are based on a -2 log likelihood test; ^c^Accessibility Index of Australia; ^d^1 indicates the lowest 10% of the population in the state and 4 indicates the highest 30% of the population in the state; **Abbreviations:** SE, Standard Error; HT, Hormone Therapy; FFDM, Full Field Digital Mammograms

**Table S3:** Regression results for absolute dense area among Aboriginal (n=387) and non-Aboriginal (n=430) women with FFDM mammograms where SEIFA and ARIA scores are based on full residential address.

| Absolute Dense Area (cm^2^)^a^ | Aboriginal (n=387) | | | | Non-Aboriginal (n=430) | | | |
| --- | --- | --- | --- | --- | --- | --- | --- | --- |
| Characteristic | Univariate | | Multivariate | | Univariate | | Multivariate | |
|  | β (SE) | P-value^b^ | β (SE) | P-value^b^ | β (SE) | P-value^b^ | β (SE) | P-value^b^ |
| Age at mammogram (per year) | -0.076 (0.012) | <0.001 | **-0.060 (0.011)** | **<0.001** | -0.080 (0.011) | <0.001 | **-0.054 (0.0099)** | **<0.001** |
| Non-dense area (per cm^2^) | -0.019 (0.0019) | <0.001 | **-0.018 (0.0019)** | **<0.001** | -0.021 (0.0017) | <0.001 | **-0.018 (0.0017)** | **<0.001** |
| HT use in the last 12 months | 0.25 (0.37) | 0.497 |  |  | 0.031 (0.25) | 0.902 |  |  |
| Family history of breast cancer | 0.74 (0.42) | 0.081 | **0.74 (0.36)** | **0.044** | 0.18 (0.35) | 0.598 | **0.18 (0.29)** | **0.536** |
| ARIA^c^ |  | 0.387 |  | 0.746 |  | 0.033 |  | **0.077** |
| Major city | Reference |  | Reference |  | Reference |  | **Reference** |  |
| Inner and outer regional | 0.27 (0.33) |  | 0.19 (0.28) |  | -0.26 (0.27) |  | **-0.27 (0.23)** |  |
| Remote | 0.35 (0.32) |  | 0.23 (0.27) |  | 0.41 (0.27) |  | **0.27 (0.23)** |  |
| Very remote | -0.10 (0.28) |  | 0.014 (0.24) |  | -0.35 (0.28) |  | **-0.25 (0.23)** |  |
| Advantage and disadvantage index^d,e^ |  | 0.317 |  | 0.319 |  | 0.015 |  | 0.155 |
| 1 (lowest) | Reference |  | Reference |  | Reference |  | Reference |  |
| 2 | 0.33 (0.25) |  | 0.35 (0.21) |  | 0.27 (0.26) |  | 0.17 (0.22) |  |
| 3 | 0.47 (0.31) |  | 0.28 (0.26) |  | 0.70 (0.28) |  | 0.42 (0.24) |  |
| 4 (highest) | 0.51 (0.51) |  | -0.12 (0.44) |  | 0.87 (0.32) |  | 0.53 (0.27) |  |
| Disadvantage index^d,f^ |  | 0.015 |  | 0.031 |  | 0.117 |  | 0.350 |
| 1 (lowest) | Reference |  | Reference |  | Reference |  | Reference |  |
| 2 | 0.58 (0.26) |  | 0.56 (0.22) |  | 0.17 (0.26) |  | 0.10 (0.22) |  |
| 3 | 0.73 (0.29) |  | 0.55 (0.25) |  | 0.44 (0.28) |  | 0.30 (0.23) |  |
| 4 (highest) | 1.0 (0.55) |  | 0.35 (0.47) |  | 0.69 (0.32) |  | 0.41 (0.27) |  |
| Economic resources index^d,g^ |  | 0.010 |  | **0.018** |  | 0.202 |  | 0.434 |
| 1 (lowest) | Reference |  | **Reference** |  | Reference |  | Reference |  |
| 2 | 0.66 (0.25) |  | **0.61 (0.21)** |  | 0.42 (0.27) |  | 0.32 (0.22) |  |
| 3 | 0.84 (0.32) |  | **0.55 (0.27)** |  | 0.58 (0.28) |  | 0.27 (0.24) |  |
| 4 (highest) | 0.71 (0.43) |  | **0.095 (0.37)** |  | 0.36 (0.33) |  | 0.045 (0.28) |  |
| Education and occupation index^d,h^ |  | 0.594 |  | 0.847 |  | 0.002 |  | 0.103 |
| 1 (lowest) | Reference |  | Reference |  | Reference |  | Reference |  |
| 2 | 0.29 (0.26) |  | 0.17 (0.22) |  | 0.39 (0.31) |  | 0.17 (0.27) |  |
| 3 | 0.26 (0.31) |  | 0.15 (0.26) |  | 0.44 (0.31) |  | 0.34 (0.27) |  |
| 4 (highest) | 0.48 (0.45) |  | 0.25 (0.39) |  | 1.2 (0.34) |  | 0.65 (0.29) |  |

NOTE: SEIFA and ARIA indices were considered one at a time in all multivariate analyses to avoid collinearity. Effect measures in bold were included in final model. Other effect measures were adjusted for age at mammogram, non-dense area and family history for both Aboriginal and non-Aboriginal women. ^a^Square root transformed; ^b^p values are based on a -2 log likelihood test; ^c^Accessibility Index of Australia; ^d^1 indicates the lowest 10% of the population in the state and 4 indicates the highest 30% of the population in the state; **Abbreviations:** SE, Standard Error; HT, Hormone Therapy; FFDM, Full Field Digital Mammograms

**Table S4:** Regression results for percentage dense area among Aboriginal (n=387) and non-Aboriginal (n=430) women with FFDM mammograms where SEIFA and ARIA scores are based on full residential address.

| Percentage dense area (%)^a^ | Aboriginal (n=387) | | | | Non-Aboriginal (n=430) | | | |
| --- | --- | --- | --- | --- | --- | --- | --- | --- |
| Characteristic | Univariate | | Multivariate | | Univariate | | Multivariate | |
|  | β (SE) | P-value^b^ | β (SE) | P-value^b^ | β (SE) | P-value^b^ | β (SE) | P-value^b^ |
| Age at mammogram (per year) | -0.070 (0.011) | <0.001 | **-0.050 (0.0084)** | **<0.001** | -0.089 (0.011) | <0.001 | **-0.046 (0.0072)** | **<0.001** |
| Non-dense area (per cm^2^) | -0.025 (0.0016) | <0.001 | **-0.024 (0.0015)** | **<0.001** | -0.032 (0.0013) | <0.001 | **-0.029 (0.0013)** | **<0.001** |
| HT use in the last 12 months | 0.44 (0.34) | 0.198 |  |  | 0.0080 (0.25) | 0.974 |  |  |
| Family history of breast cancer | 0.40 (0.39) | 0.304 |  |  | 0.12 (0.35) | 0.739 |  |  |
| ARIA^c^ |  | 0.358 |  | 0.688 |  | 0.049 |  | 0.109 |
| Major city | Reference |  | Reference |  | Reference |  | Reference |  |
| Inner and outer regional | 0.20 (0.30) |  | 0.11 (0.22) |  | -0.24 (0.27) |  | -0.17 (0.17) |  |
| Remote | 0.18 (0.29) |  | 0.048 (0.22) |  | 0.37 (0.27) |  | 0.18 (0.17) |  |
| Very remote | -0.23 (0.26) |  | -0.12 (0.19) |  | -0.35 (0.27) |  | -0.17 (0.17) |  |
| Advantage and disadvantage index^d,e^ |  | 0.167 |  | 0.091 |  | 0.007 |  | 0.087 |
| 1 (lowest) | Reference |  | Reference |  | Reference |  | Reference |  |
| 2 | 0.35 (0.23) |  | 0.39 (0.17) |  | 0.41 (0.26) |  | 0.17 (0.16) |  |
| 3 | 0.53 (0.28) |  | 0.33 (0.21) |  | 0.80 (0.28) |  | 0.37 (0.17) |  |
| 4 (highest) | 0.58 (0.47) |  | -0.024 (0.35) |  | 0.93 (0.32) |  | 0.41 (0.20) |  |
| Disadvantage index^d,f^ |  | 0.007 |  | 0.005 |  | 0.067 |  | 0.156 |
| 1 (lowest) | Reference |  | Reference |  | Reference |  | Reference |  |
| 2 | 0.59 (0.24) |  | 0.56 (0.17) |  | 0.61 (0.26) |  | 0.15 (0.16) |  |
| 3 | 0.66 (0.27) |  | 0.49 (0.20) |  | 0.55 (0.27) |  | 0.33 (0.17) |  |
| 4 (highest) | 1.1 (0.51) |  | 0.30 (0.37) |  | 0.76 (0.31) |  | 0.36 (0.19) |  |
| Economic resources index^d,g^ |  | 0.010 |  | **0.003** |  | 0.120 |  | 0.517 |
| 1 (lowest) | Reference |  | **Reference** |  | Reference |  | Reference |  |
| 2 | 0.58 (0.23) |  | **0.61 (0.17)** |  | 0.41 (0.26) |  | 0.23 (0.16) |  |
| 3 | 0.75 (0.29) |  | **0.43 (0.22)** |  | 0.64 (0.28) |  | 0.19 (0.17) |  |
| 4 (highest) | 0.82 (0.40) |  | **0.15 (0.29)** |  | 0.54 (0.33) |  | 0.094 (0.20) |  |
| Education and occupation index^d,h^ |  | 0.439 |  | 0.732 |  | <0.001 |  | **0.073** |
| 1 (lowest) | Reference |  | Reference |  | Reference |  | **Reference** |  |
| 2 | 0.27 (0.24) |  | 0.14 (0.18) |  | 0.56 (0.31) |  | **0.16 (0.19)** |  |
| 3 | 0.38 (0.28) |  | 0.22 (0.21) |  | 0.53 (0.31) |  | **0.32 (0.19)** |  |
| 4 (highest) | 0.49 (0.42) |  | 0.17 (0.31) |  | 1.4 (0.34) |  | **0.50 (0.21)** |  |

NOTE: SEIFA and ARIA indices were considered one at a time in all multivariate analyses to avoid collinearity. Effect measures in bold were included in final model. Other effect measures were adjusted for age at mammogram and non-dense area. ^a^Square root transformed; ^b^p values are based on a -2 log likelihood test; ^c^Accessibility Index of Australia; ^d^1 indicates the lowest 10% of the population in the state and 4 indicates the highest 30% of the population in the state; **Abbreviations:** SE, Standard Error; HT, Hormone Therapy
